# Supercritical water oxidation of phenol and process enhancement with in situ formed Fe_2_O_3_ nano catalyst

**DOI:** 10.1007/s11356-021-16390-0

**Published:** 2021-09-24

**Authors:** Ammar Al-Atta, Farooq Sher, Abu Hazafa, Ayesha Zafar, Hafiz M. N. Iqbal, Emina Karahmet, Edward Lester

**Affiliations:** 1 Department of Chemical and Environmental Engineering, University of Nottingham, University Park, Nottingham, NG7 2RD UK; 2 Oil and Gas Refinery Department, Al-Farabi University College, Baghdad, Iraq; 3 Department of Engineering, School of Science and Technology, Nottingham Trent University, Nottingham, NG11 8NS UK; 4 International Society of Engineering Science and Technology, Nottingham, UK; 5 Department of Biochemistry, University of Agriculture, Faisalabad, 38040 Pakistan; 6 Institute of Biochemistry and Biotechnology, Faculty of Biosciences, University of Veterinary and Animal Sciences, Lahore, Pakistan; 7 Tecnologico de Monterrey, School of Engineering and Sciences, 64849 Monterrey, Mexico; 8 Department of Biochemistry, Faculty of Pharmacy, University of Modern Science, 88000 Mostar, Bosnia and Herzegovina

**Keywords:** Environmental management, Supercritical water oxidation, Nanoparticles, Fenton reactions, Hematite, Phenol, Counter current mixing reactor, Wastewater treatment

## Abstract

During the past few decades, the treatment of hazardous waste and toxic phenolic compounds has become a major issue in the pharmaceutical, gas/oil, dying, and chemical industries. Considering polymerization and oxidation of phenolic compounds, supercritical water oxidation (SCWO) has gained special attention. The present study objective was to synthesize a novel in situ Fe_2_O_3_nano-catalyst in a counter-current mixing reactor by supercritical water oxidation (SCWO) method to evaluate the phenol oxidation and COD reduction at different operation conditions like oxidant ratios and concentrations. Synthesized nano-catalyst was characterized by powder X-ray diffraction (XRD) and transmission electron microscope (TEM). TEM results revealed the maximum average particle size of 26.18 and 16.20 nm for preheated and non-preheated oxidant configuration, respectively. XRD showed the clear peaks of hematite at a 2θ value of 24, 33, 35.5, 49.5, 54, 62, and 64 for both catalysts treated preheated and non-preheated oxidant configurations. The maximum COD reduction and phenol oxidation of about 93.5% and 99.9% were observed at an oxidant ratio of 1.5, 0.75 s, 25 MPa, and 380 °C with a non-preheated H_2_O_2_ oxidant, while in situ formed Fe_2_O_3_nano-catalyst showed the maximum phenol oxidation of 99.9% at 0.75 s, 1.5 oxidant ratio, 25 MPa, and 380 °C. Similarly, in situ formed Fe_2_O_3_ catalyst presented the highest COD reduction of 97.8% at 40 mM phenol concentration, 1.0 oxidant ratio, 0.75 s residence time, 380 °C, and 25 MPa. It is concluded and recommended that SCWO is a feasible and cost-effective alternative method for the destruction of contaminants in water which showed the complete conversion of phenol within less than 1 s and 1.5 oxidant ratio.

## Introduction

During the past few decades, wastewater purification and treatment has gained special attention due to global water scarcity and environmental pollution. Organic wastewater is considered most hazardous due to its poor biodegradability and high salinity that comes from pharmaceutical, oil/gas, petroleum, pesticide, textile, chemical, and dyeing industries (Zhang et al. 2020b). The emerging evidence revealed that phenol and its derivates (ortho-(ethoxymethyl)-phenol, 2-methoxy-4-methyl-phenol, resorcinol, and guaiacol) are ranked as one of the highly toxic organic compounds globally by the United States Environmental Protection Agency (USEPA) due to their several health concerns including carcinogenic and teratogenic effects (Ren et al. 2018, Ren et al. 2017). However, the concentration of phenolic waste continues to increase in response to accelerated urbanization and industrialization. Therefore, some physiochemical techniques are required to remove organic content and break a ring-shaped phenol structure from wastewater (Zhang et al. 2020a).

Traditionally different strategies such as coagulation, adsorption, membrane separation, and incineration methods have been employed for the treatment of toxic and hazardous wastewater to remove organic and inorganic pollutants. These techniques were used to destroy hazardous waste or convert them into non-hazardous materials such as antacids. However, all of these techniques are limited to desire outcomes due to their high cost; hazardous stack gas emissions such as NOx, dioxin, and furan, low concentration waste, relatively longer residence time, and reactor volumes (Fang & Xu 2014, Lin & Ma 2012). The conventionally most applied microbial method is also limited to phenolic wastewater treatment due to the delocalization of π bond in the phenolic ring. Nevertheless, long-term discharge and treatment difficulties of phenolic wastewater could lead to more discharge of industrial waste on the water surface that is a serious problem for not only the human health but also the ecosystem. However, an alternative strategy should be adopted to treat phenolic wastewater (Pillai & Gupta 2016, Zhang et al. 2019).

During last few years, supercritical water oxidation (SCWO) technique has gained extensive consideration in the treatment of organic wastewater because of high ion mass, low dielectric constant, and low density. Accumulated data revealed that SCW used oxidant gases (O_2_, O_3_, and NO_2_), non-polar properties of water above its critical point (22.1 MPa and 374 °C), and non-polar hydrocarbons to develop a single phase. SCWO has advantages over conventional reported waste treatment techniques due to its efficiency and environmentally friendly behavior (Kıpçak & Akgün 2012, Zhao et al. 2020). The destruction of organic compounds in SCWO is primarily based upon radical reaction mechanisms rather than ionic reactions due to very low values of the ionic product of water at supercritical conditions. The organic molecules are attacked by free radicals which are produced during the oxidation of organic compounds in SCW (see Eqs. (1–3) (Fang & Xu 2014)).

Hydroxyl generation:

1 $${\mathrm{H}}_2{\mathrm{O}}_2\to 2\mathrm{H}{\mathrm{O}}^{\cdotp }$$

Hydrogen removal and generation of organic radical (R^·^):

2 $$\mathrm{RH}+\mathrm{H}{\mathrm{O}}^{\cdotp}\to {\mathrm{R}}^{\cdotp }+{\mathrm{H}}_2\mathrm{O}$$

Production of peroxy radical (ROO^·^):

3 $${\mathrm{R}}^{\cdotp }+{\mathrm{O}}_2\to {\mathrm{R}\mathrm{OO}}^{\cdotp }$$

Peroxy radical (ROO^·^) extracts a hydrogen atom to form an organic peroxide (ROOH) and a new organic radical (R^·^). Generally, different oxidant including air, O_2_, and H_2_O_2_ are applied into the superheated water during supercritical water oxidation (SCWO) that not only increase the operational cost but also enhance energy demand (500–700 °C) (Huelsman and Savage, 2013, Zhang et al. 2020a). However, the proper usage of catalysts could effectively improve the oxidant concentration and reactor energy demand without affecting the operational performance (Xu et al. 2020). An appropriate catalyst not only improves the removal of chemical oxygen demand (COD) of the waste but also enhances the selectivity of phenols into CO_2_, CH_4_, and H_2_(Nadjiba et al. 2017). Recently, different heterogeneous catalysts including V_2_O_5_/Al_2_O_3_, MnO_2_/CeO_2_, TiO_2_, Caroline 150, CuO/ZnO/CoO, and MnO_2_ have been used by different researchers for phenol oxidation (Abdpour and Santos, 2020, De Silva et al. 2017, Huelsman and Savage, 2013).

Top et al. (2020) reported that supercritical water oxidation (SCWO) process significantly removed over 90% COD, TOC, SS, and BOD of real hospital wastewater after 60 s retention time at 25 ± 1 MPa, 450 °C, and H_2_O_2_/COD ratio of 1:1. They also observed over 90 and 80% removal of phosphorus and phenol respectively at the same reaction condition. Li et al. (2020) stated that the SCWO method with MnO_2_/CeO_2_ catalyst showed about 98.52% COD and 67.18% NH_3_–N removal from semi-coke wastewater at 2 min retention time, 1.3 oxidation coefficient, 25 MPa, and 550 °C. Various studies on SCWO have been done over the past few decades, but their uses in industry are still in their infancy. The scale-up commercialization development of the procedure is subject to problems associated with operational cost presented by high energy input required to reach optimal reaction temperature (550–750°C), although extensive research had been devoted to examine the optimization of reaction considerations like oxidant concentration, temperature, and retention time. However, very little study has been made on mixing and reactor geometry. Therefore, there is a need to conduct an extensive study on the selective oxidation of para-xylene in supercritical water under the consideration of reactor configuration to control product distribution (Pérez et al. 2016).

The formulation of metal oxide nanoparticles by supercritical water oxidation is one of the rapid green methods due to quick and continuous mixing of supercritical water stream with a cold metal salts solution stream in reactor volume. Different reactor geometries had been used to mix these two fluids, starting from very basic T- and Y-shaped, tube-in-tube configurations to more complex mechanical hybrid designs (Lester et al. 2018). The tube-in-tube counter-current mixing design uses the natural convection that arises from the difference of densities between supercritical water and the cold flow to creates efficient mixing between reagents (Lester et al. 2006). Using a counter-current mixing reactor for the SCWO process can offer the following advantages: (1) cool feed injection of organic compounds may prevent damage of parts and/or piercing used to heat feed due to hetero atoms that may be present in the organic compound stream causing severe corrosion to the equipment. (2) Supercritical water inside reactor contacts with organic compound by immediate warming. Fast heating may avoid charring or organic pyrolysis compounds formation. (3) Strong downstream eddies may prevent any particle accumulation on the reactor surface. (4) Oxidation rate through catalytic activities could be speedup by the synergic effect of metal oxide and organic material oxidation. (5) A mobile, large surface area is provided by nanoparticles so that any salt present in the waste stream will more likely adhere to the particle instead of reactor walls or other components.

Fenton reactions have been widely used as an advanced oxidation process for the destruction of toxic and hazardous organic chemicals. The combination of Fenton reagents (iron salt and hydrogen peroxide) produces a highly oxidative agent of hydroxyl radicals (HO^·^). The organic molecules are easily attacked by hydroxyl radicals which are produced during the Fenton reactions. The present study results indicated that the addition of a small amount of dissolved iron salt to H_2_O_2_ solution that might increase the performance of SCWO on the degradation of acrylic acid, which play a synergistic role in the Fenton oxidation and SCWO at relatively moderate reaction temperature (Al-Atta et al. 2018).

In the present study, the combination of supercritical water oxidation (SCWO) with Fenton oxidation was used for the formulation of in situ Fe_2_O_3_ nano-catalyst in a counter-current mixing reactor for the oxidation of phenol at different operational situations including, oxidant ratio, temperature, residence time, and feed concentration. Furthermore, the study also explained the effect of non-preheated and catalytic non-preheated oxidants on the destruction of an organic compound like phenol in a counter-current mixing reactor.

## Materials and methods

### Chemicals

Phenol (≥99%, C_6_H_6_O) was obtained from Sigma Aldrich, USA. H_2_O_2 _(30% w/v) was purchased from Fisher. Iron (III) nitrate nonahydrate (≥99%, Fe(NO_3_)_3_·9H_2_O) got from Acros Organics. Feed solutions of various concentrations were made from deionized and distilled water.

### Counter current mixing reactor setup

**Fig. 1:**
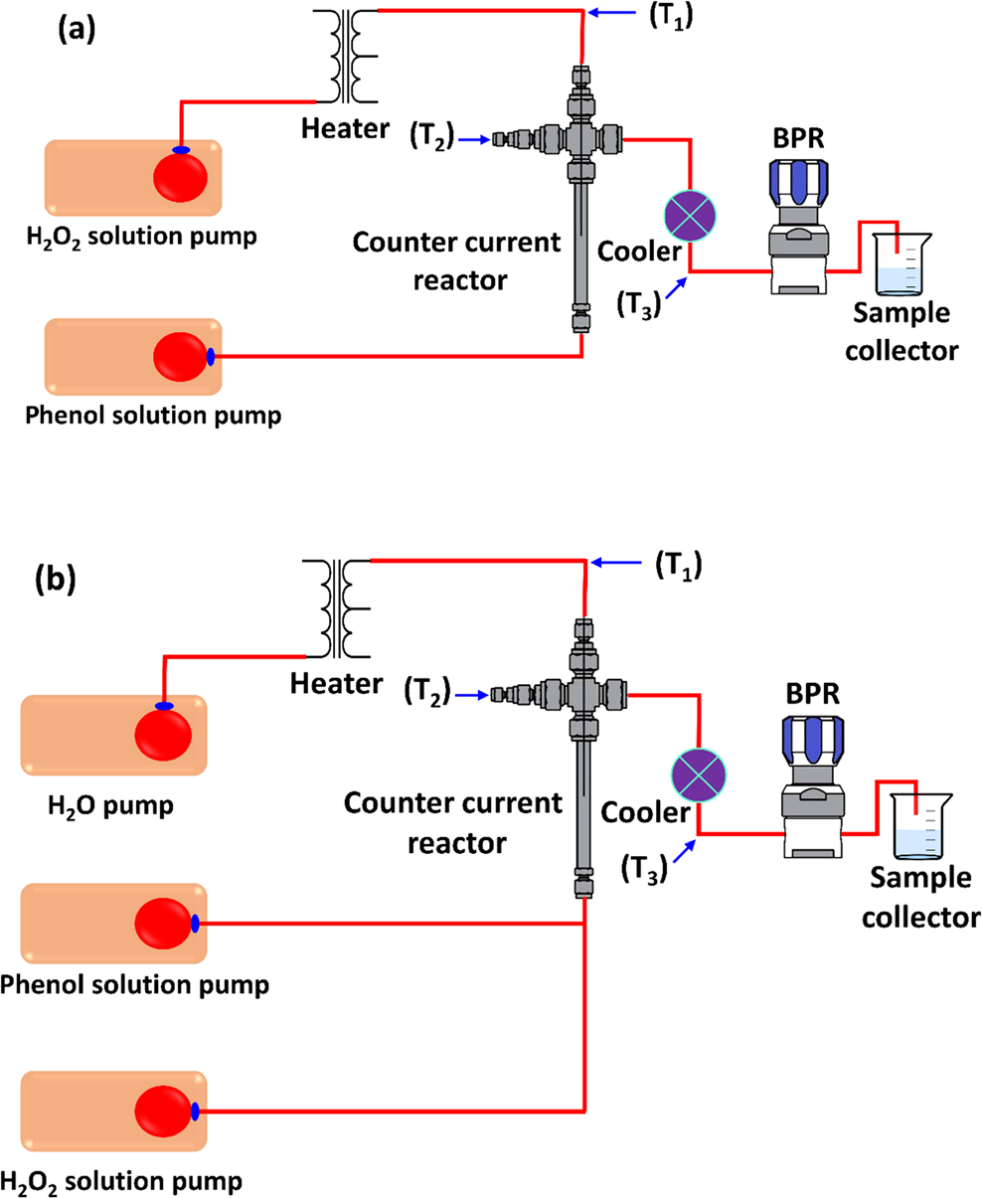
The schematic diagram of counter current mixed reactor with supercritical water oxidation process for **a** preheated and **b** non-preheated configuration. T_1_ measures immediately after the heater. T_2_ is the post mixed flow. T_3_ is the temperature prior to the back-pressure regulator

The equipment setup (counter-current mixing reactor) was used to conduct the oxidation experimental work for the continuous production of nanoparticles (see Fig. 1). 316 stainless steel (Swagelok) was used to make tubing, fitting, and counter current reactor. Temperature and pressure in the system were preserved by a back-pressure regulator (BPR Pressure Tech, UK) and using Picolog software connected to a personal computer, respectively. The reaction volume was projected from the mixing point to the outlet of the reactor and found to be 1.156 cm^3^.

For the supercritical water oxidation experiment, an organic material solution (phenol, C_6_H_6_O) was introduced to the system at a pre-set flow rate of up to 12 mL/min, using a Gilson HPLC (30×) pump equipped with a 25-silicon carbon (SC) pump head. The stream rolled through a check valve before entering the counter-current reactor from the inlet at the base as an upward flow without any preheating. Meanwhile, a distilled water stream that may or not contain H_2_O_2_ was pumped into the system with a maximum flow of 24 mL/min using a Gilson HPLC pump. This then proceeded to flow through a check valve before owing through a pressure relief valve and piezoelectric pressure transducer, which in turn was connected to a digital pressure monitor. The stream tossed through an analog pressure gauge before entering a 6 m length of 1/4″ tubing coiled around a 2 KW electric heater. A thermocouple within the heater block acted as feedback control. After the heater, the water feed flowed past a thermocouple before entering the top of the reactor as a downflow (De Silva et al. 2017).

Upon exiting the reactor, the oxidized product like oxygen and carbon dioxide developed during the oxidation process was partially cooled by a primary vertical cooling loop. The products then flowed through a Tee-shaped union at which a thermocouple was mounted on the perpendicular port, allowing the initial cooling temperature to be monitored. The product was subsequently cooled by a counter-current heat exchanger which brought the temperature down to ambient conditions approximately (Al-Atta et al. 2020, Huddle et al. 2017).

### Oxidant configuration assessment of phenol

Initial trails have been made to assess whether or not the reactor configuration was suitable for the entire degradation of organic content of phenol in supercritical water in terms of oxidative efficiency. Both uncatalyzed and catalyzed (Fe_2_O_3_) reactions for non-preheated and preheated oxidant configurations were performed. The experimental setup for non-preheated and preheated oxidant configurations is represented in Fig. 1. The catalytic reactions were obtained by injecting iron nitrate into the reactor as up-flow along with phenol solution. The initial experiments were conducted at 380 °C, 25 MPa, 6.6–7.4 g/L COD feed, 20 mM catalyst (Fe(NO_3_)_3_·9H_2_O), 1.5 oxidant ratio, and 0.75 s residence time.

**Fig. 2:**
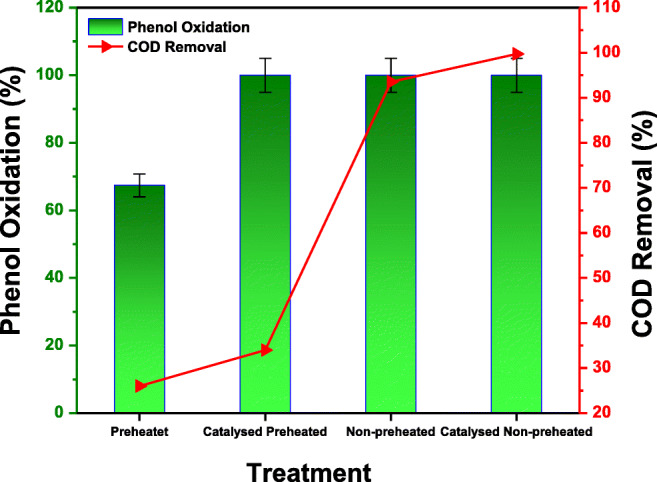
Preliminary test outcomes for phenol oxidation and COD reduction at different treatments

The reaction temperature was set to exceed the critical point of water which is governed by heater limitation. A short residence time was chosen because it was shown to have very little effect on the oxidation of other compounds like acrylic acid (Al-Atta et al. 2018). The results of the preliminary experiments in terms of the removal of phenol and COD are shown in Fig. 2. Although a high phenol conversion was obtained in the catalytic reaction, the COD removal of uncatalyzed and catalyzed preheated oxidant scenarios at excess oxygen was 26 and 34% respectively. Therefore, it was decided that these configurations are not suitable for the complete oxidation of phenol. Consequently, all subsequent experiments were conducted using the uncatalyzed and catalyzed non-preheated oxidant configurations.

#### Non-preheated oxidant configuration

The influence of different oxidant ratios (0.0–2.0) was examined under the presence of different oxidant concentrations. The phenol was supplied at a constant concentration of 5 mM at reaction conditions which corresponded to 112.2 mM or COD of 6.6–7.4 at the feedstock. The operating conditions were maintained at 380 °C, 25:0 MPa, and a residence time of 0.75 s. The total ion current traces (TICs) for phenol samples were also examined at an oxidant ratio of 1.0 and 1.5.

#### Catalytic non-preheated oxidant configuration

Hematite (Fe_2_O_3_) was chosen as a nano-catalyst for phenol oxidation in supercritical water. Iron (III) nitrate nonahydrate (Fe(NO_3_)_3_·9H_2_O) was used as a precursor in the production of hematite. In a non-preheated oxidant configuration, supercritical water was mixed with a mixture of phenol, Fe(NO_3_)_3_·9H_2_O, and H_2_O_2_ solution. The in situ formed Fe_2_O_3_ catalyst could enhance the oxidation of organic materials. Like non-preheated oxidant configuration, the influence of oxidant ratio on the phenol and COD removal was investigated at 25 MPa, 380 °C, and Fe(NO_3_)_3_·9H_2_O concentration of 10 mM (Al-Atta et al. 2018).

### Chemical oxygen demand reduction

Chemical oxygen demand (COD) of organic content (phenol) in water samples was observed for every trial to investigate the influence of changing various operational parameters on the removal of COD. The COD experiment included oxidizing the liquid samples’ organic content under acidic conditions at 148 °C for 2 h. Strong oxidizing agents of potassium dichromate and sulfuric acid oxidized the organic matter in the presence of silver sulfate as a catalyst. These compounds are all present in the sample cuvettes LCI400 (HACH LANGE LTD, Manchester, UK) used for COD measurement. Two milliliters of organic compound samples in COD cuvettes were digested in a LT 200 COD reactor. The end products were water, and carbon dioxide (Lee et al. 2011, Wu and Englehardt, 2012). As the cuvette rotated, it was measured 10 times within 5 s for an average value that eliminates any abnormal results. The reduction in COD was calculated using Eq. (4) (Wu & Englehardt 2012).

4 $$\mathrm{COD}\ \mathrm{reduction}\ \left(\%\right)=\frac{{\mathrm{COD}}_{\mathrm{initial}}-{\mathrm{COD}}_{\mathrm{final}}}{{\mathrm{COD}}_{\mathrm{final}}}\times 100$$

Two separated streams of distilled water and a known concentration of phenol and two parted streams of distilled water in a ratio of 2:1 were poured through the experimental rig at the start of operation without any preheating or pressurizing. The subsequent mixed solution flow was additionally diluted to a factor of 0.2. Catalyst characterization. The powdered X-ray diffraction (XRD) analysis technique was applied to determine the phase composition, crystal clear size, and structure of a solid sample. The XRD analysis was examined using a Bruker D8 Advance system (Bruker AXS, Germany) through Cu Kα radiation (λ=1.54056 A) in a 2θ range between 15° and 75°. The Scherrer method, assuming Gaussian peak broadening, was used to calculate the crystallite size of metal oxide nanoparticles (Abdpour and Santos, 2020, Al-Atta et al. 2018).

Transmission Electron Microscopy (TEM; Philips Tecnai G220) analysis was used to determine particle size and morphology based on the contrast difference of the electrons that have been transmitted through. TEM analysis was used for the characterization of metal oxide nanoparticles produced through the oxidation of phenol in supercritical water. Nanometal oxides produced in water from the oxidation experiments were allowed to settle for 24 h. A few drops of settled nanoparticles were then sampled and suspended in acetone for examination by TEM. The JEOL 2100F system (FEGTEM) was used for TEM images operating at an acceleration voltage of 100 kV (Meng et al. 2018).

## Results and discussion

### Morphological and crystalline assessment of catalyst

**Fig. 3:**
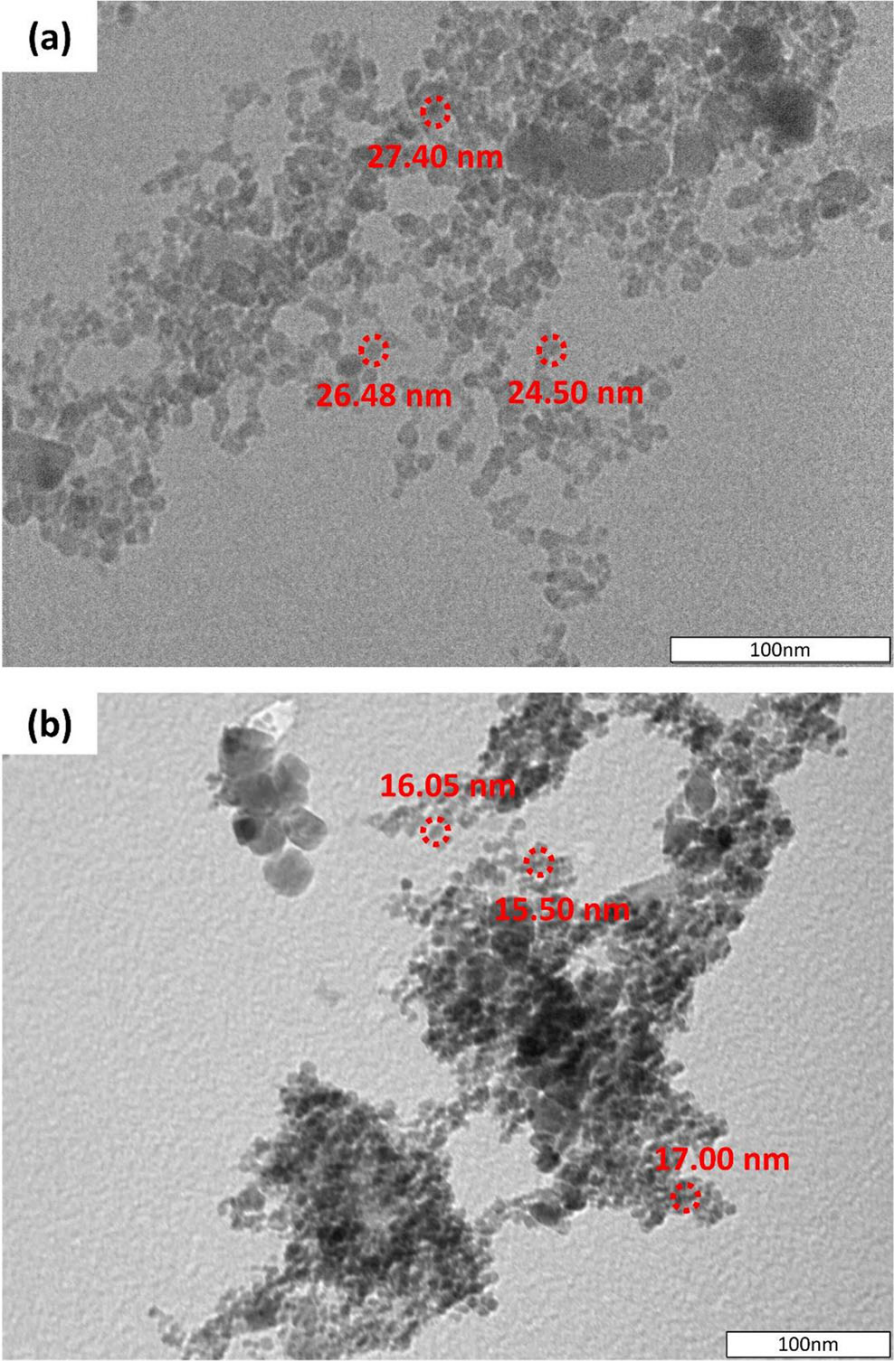
TEM images of Fe_2_O_3_ nanoparticles obtained from **a** preheated oxidant and **b** non-preheated oxidant configuration

Figure 3 showed the particle size and morphology results of hematite suspension for the non-preheated and preheated oxidant configuration at a precursor concentration of 20 mM. The morphology was observed to be spherical with detectable edges. The average particle size using ImageJ software was determined as 16.20 and 26.18 nm for the non-preheated and preheated oxidant configuration, respectively. The average particle size was gradually decreased with increasing the Fe compound that was due to the molar ratio of Fe/O. Moreover, Fe/O results in more pore volume and small pore size (Zhang et al. 2018). Salari (2019) observed that TEM images of Fe_2_O_3_ were regular in shape with an average size of 15 nm.

**Fig. 4:**
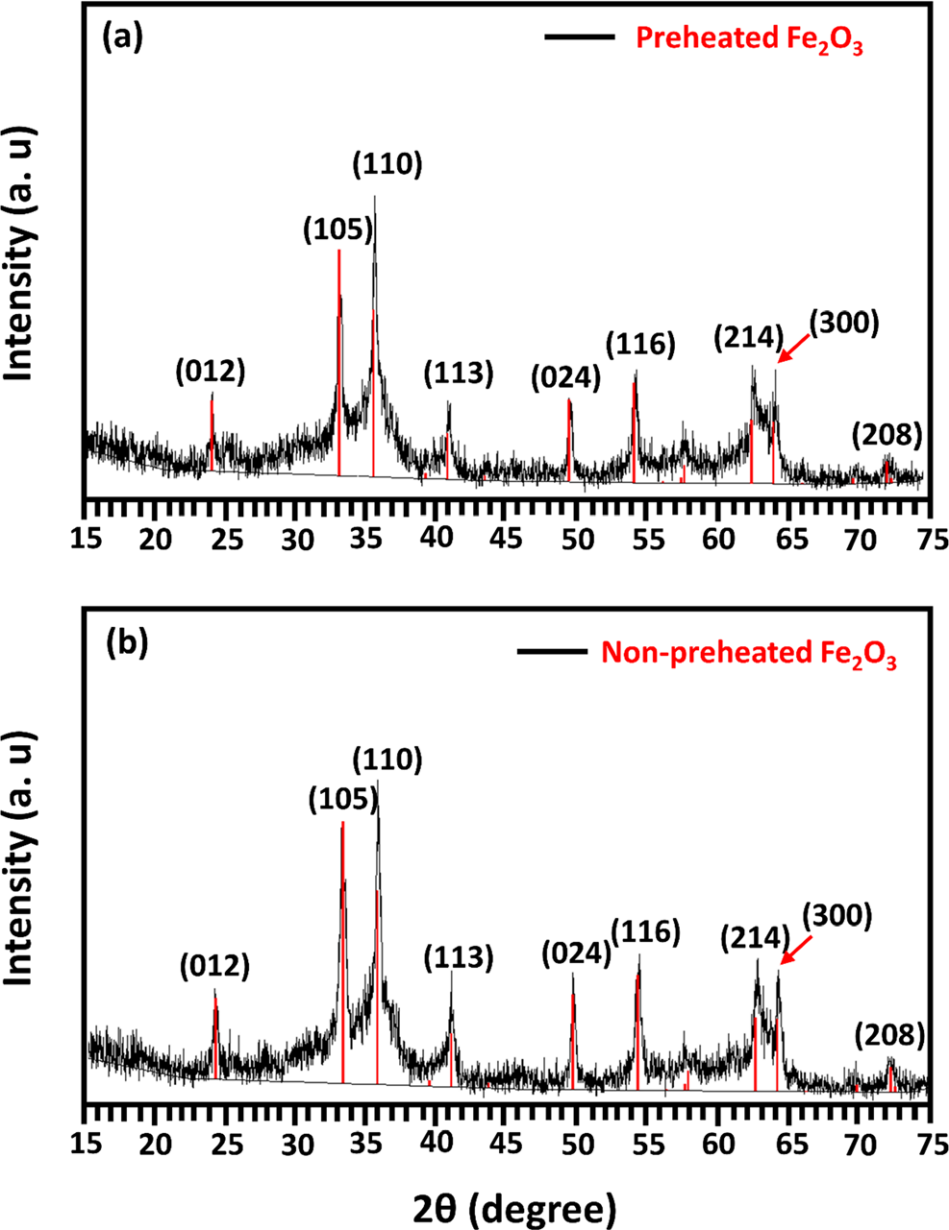
XRD patterns of hematite nanoparticles for (**a**) preheated oxidant and (**b**) non-preheated oxidant scenario obtained at 380 °C, 25 MPa, oxidant ratio of 1.5 and 20 mM of metal salt concentration

Figure 4 reports the XRD results of hematite suspension for non-preheated and preheated oxidant configurations. XRD patterns confirmed the presence of the crystalline phase of hematite. The Scherrer equation was utilized for crystallite diameter determinations that were noted as 25.5 and 21.8 nm for the preheated and non-preheated oxidant configurations, respectively. Results showed the clear peaks of hematite at 2 theta values of 24, 33, 35.5, 49.5, 54, 62, and 64 for both the catalyst preheated and non-preheated oxidant configurations. There was no specific difference between hematite (Fe_2_O_3_) suspension for non-preheated and preheated oxidant configuration. The present study results are in correlation with the findings of Salari (2019), who stated that diffraction peaks that appeared at 2θ values of 24, 33, 35, 49, 54, 62, and 64 related to 012, 110, 113, 024, 214, and 300 planes respectively are due to Fe_2_O_3_. Similarly, Zangeneh Kamali et al., 2014 reported that XRD peaks appearing at 2 theta values of 24 (012), 35 (113), 41 (202), 54 (116), and 62 (214) are due to nanocomposites of α-Fe_2_O_3_. The  lack of any diffraction peak of Fe(OH)_3_ and Fe(OH)_2_ indicated that Fe-based SCW had been completely converted into Fe_2_O_3_.

### COD reduction and phenol oxidation in non-preheated oxidant configuration

**Fig. 5:**
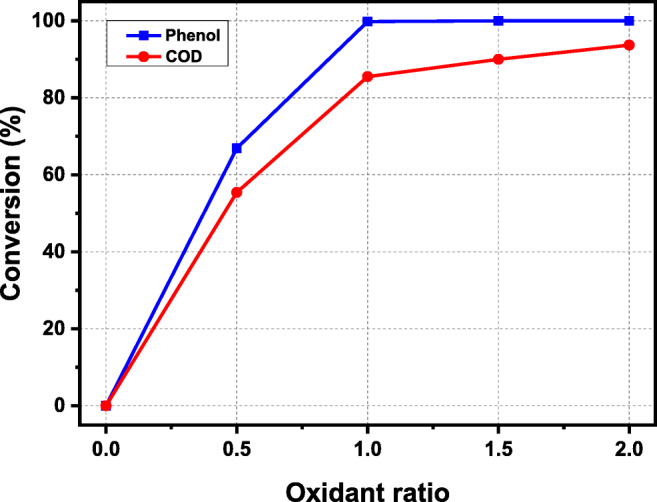
Effect of oxidant ratio of non-preheated configuration on phenol oxidation and COD removal at 380 °C, 25 MPa, residence time of 0.75 s, and an initial phenol concentration of 5 mM

The results of the effect of oxidant concentration in the non-preheated oxidant configuration for phenol and COD are given in Fig. 5. Results showed that hydrolysis reaction has no effect on phenol degradation. However, when the concentration of oxidant increased to the stoichiometric amount, complete elimination of phenol was obtained. COD reduction increased by approximately 94% at an oxidant ratio of 2.0, which presented a great reaction dependence on HO concentration. At the highest oxidant ratio, the close values of phenol and COD removal indicate that the free radical HO reduced the formation of undesirable intermediates.

Jarana and co-workers observed about 71% phenol reduction at a reactor temperature of 390 °C, the pressure of 25 MPa, the oxidant ratio of 4.8, and residence time of 104 s (Jarana et al. 2010). Yan et al. (2020) stated that SCWO treatment significantly showed the COD reduction efficiency of sludge and p-tert-butylcatechol (TBC) of about 77 and 89% at oxidant ratio of 1, and 98 and 99% at oxidant ratio of 8, constant temperature of 550 °C, the pressure of 25 MPa, and residence time of 5 min. Similarly, Li et al. (2019) reported that SCWO treatment of dying sludge showed up to 99.80% COD reduction at a reactor temperature of 600 °C, oxidation coefficient of 1.2, the pressure of 25 MPa, and residence time of 600 s.

Compared to other research works on the oxidation of phenol in supercritical water, the non-preheated oxidant configuration exhibits a high reduction of organic content at a relatively mild temperature and short reaction time. This is because the properties of counter-current mixing reactor provide instant and rapid heating of the organic compounds and oxidant. This fast heating of the organic compounds could avoid the formation of organic pyrolysis intermediates which are more difficult to oxidize in the reaction zone.

### Effect of catalyst in non-preheated configuration

**Fig. 6:**
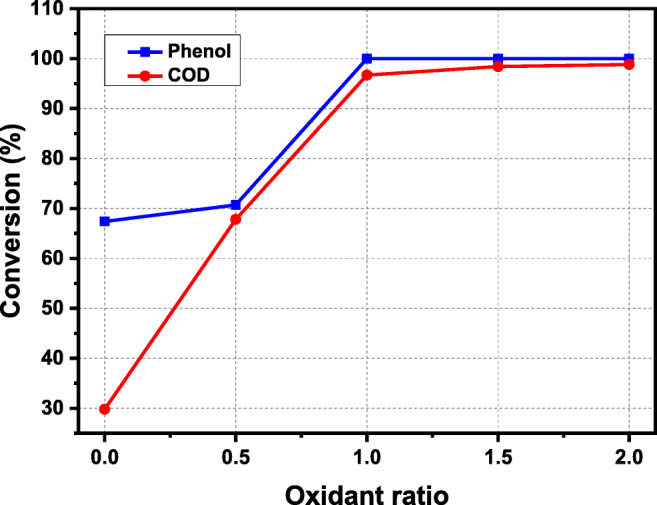
Effect of oxidant ratio of catalyst non-preheated configuration on phenol oxidation and COD removal at 380 °C, 25 MPa, residence time of 0.75 s, initial phenol concentration of 5 mM, and Fe(NO_3_)_3_·9H_2_O concentration of 10 mM

Figure 6 reports the effect oxidant ratio for the removal of COD and conversion of phenol. Results demonstrated that in the absence of an oxidant, the elimination percentage (%) of phenol and COD was 67.4 and 29.8% respectively. By increasing the concentration of oxidant to a stoichiometric amount, the reaction approaches completion for phenol and COD (see Fig. 6). The maximum COD reduction and phenol conversion were observed to be 99.9 and 99.89% respectively with an oxidant ratio of 2.0 and reactor a  temperature of 380 °C, a pressure of 25 MPa, a residence time of 0.75 s, initial phenol concentration of 5 mM, and an Fe(NO_3_)_3_·9H_2_O concentration of 10 mM. The COD removal efficiency went up rapidly in the presence of salt ions, which was due to Fe^2+^ and H_2_O_2_ constitute Fenton’s reagent, resulting in improved H_2_O_2_ decomposition into HO^·^ by having robust oxidation capacity. The findings of Ma et al. (2017) revealed that more oxygen concentration gives rise to better phenol oxidation in the catalytic reaction. They also observed almost similar values of COD removal and phenol reduction, which signifies that the catalyst lowered the formulation of detrimental intermediates.

**Fig. 7:**
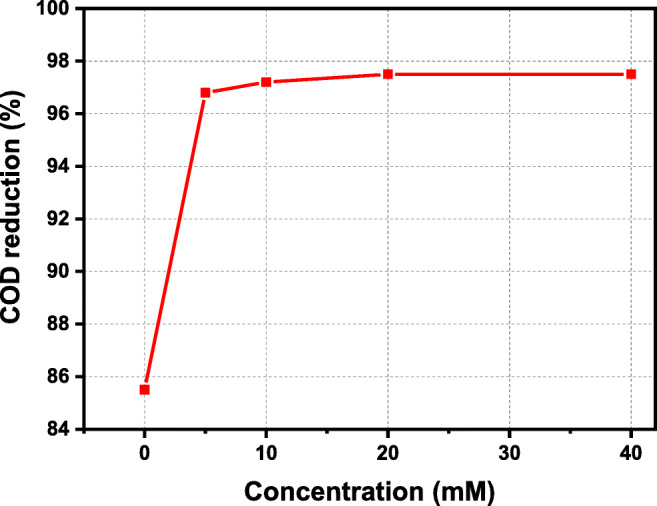
The effect of in situ formed catalyst on COD removal at different concentrations using a constant temperature of 380 °C, pressure of 25 MPa, residence time of 0.75 s, and oxidant ratio of 1.0

Figure 7 shows the effect of Fe(NO_3_)_3_·9H_2_O concentration on COD treatment. The operating temperature, pressure, and phenol concentration were the same as for the assessment of oxidant ratio gradients. The oxidant ratio was kept constant at 1.0. Results showed that COD removal was enhanced constantly with catalyst addition until the concentration of 20 mM. The maximum COD was observed as 97.8 at phenol concentration of 5 mM, and constant temperature of 380 °C, pressure of 25 MPa, residence time of 0.75 s, and oxidant ratio of 1. Further, it was noted that the accumulation of excessive volumes of catalyst did not significantly affect organic compound elimination. Catalyst appear to have the abolity to accelerate the reaction; however,  their development is limited to a certain range of concentrations.

Jarana and co-workers (2010) observed about 99.5% phenol reduction at a reactor temperature of 390 °C, pressure of 25 MPa, oxidant ratio of 4.0, residence time of 11.4 s, and in situ formed catalyst of Cr_2_O_3_–Al_2_O_3_. Li et al. (2018) examined the degradation of phenol by SCW process and observed about 80% phenol reduction efficiency at 525 °C, water density of 0.098 kg/m^3^, residence time of 60 min, and Ni/CeO_2_ value of 0.5 g. Chen et al. (2020) revealed that in situ formed KOH catalyst by SCW treatment significantly removed up to 94.6 and 51.0% COD and NH_3_–N of landfill leachate at a residence time of 20 min, pressure of 23–26 MPa, and temperature of 650 °C. Similarly, Scandelai et al. (2020) reported that SCWO/zeolite(clinoptilolite) system significantly removed 74% COD, 98% nitrate reduction, and 81% TOC of landfill leachate at 23 MPa, and 600 °C.

When evaluating the phenol catalytic oxidation rates of various researchers to those obtained in the existence of an in situ formed catalyst, a considerable rate augmentation was noted in the latter case. Greater than 99% conversion was achieved in preheated and non-preheated oxidant scenarios in the first order of magnitude shorter residence time associated with other catalytic oxidation works. The present study results indicated that the utilization of catalysts gives a smaller number of organic acid species, which suggests that catalysts are proved as efficient in the ring-opening process. However, elevated pollutant devastation proficiency might be accomplished at lower temperatures when utilizing a catalyst in the supercritical reactor system.

## Conclusions

The uncatalyzed and catalyzed non-preheated oxidant configuration was chosen to perform the SCWO of phenol. Experiments were conducted at a variety of oxidant ratios and iron nitrate concentrations. It was observed that present configurations provide superior performance to a conventional SCWO process in terms of COD removal. Almost a complete removal of phenol (99.9%) was observed at 380 °C for a residence time of less than 1 s in both configurations (non-preheated and catalyst non-preheated). Reductions in COD in excess of 94% were obtained in the non-preheated oxidant scenario. Near-complete COD removals (99.9%) were achieved at the catalytic SCWO reactions. Chromatographic information showed that only acetic acid was produced as an intermediate compound. It was determined that SCWO combined with in situ catalyst formation in  a counter-current mixing reactor resulted in COD and phenol removal efficiencies greater than other researchers' work. Although phenol is completely oxidized in different configurations, however, some recalcitrant compounds like ammonia may require rigorous reaction conditions. Therefore, two-stage operations will be useful to increase the residence time and hence the destruction efficiency of the process. However, future research should consider the identification and quantification in detail of the products and the remaining by-products in order to build a proper reaction mechanism.

## Data Availability

Not applicable.
